# Change in Cognitive Performance by Race or Ethnicity and Multimorbidity Among Older Americans

**DOI:** 10.1093/geroni/igaa057.1054

**Published:** 2020-12-16

**Authors:** Ana Quinones, Siting Chen, Anda Botoseneanu, Heather Allore, Jason Newsom, Stephen Thielke, Jeffrey Kaye

**Affiliations:** 1 Oregon Health & Science University, Portland, Oregon, United States; 2 OHSU-PSU School of Public Health, Portland, Oregon, United States; 3 University of Michigan, Dearborn, Michigan, United States; 4 Yale University, New Haven, Connecticut, United States; 5 Portland State University, Portland, Oregon, United States; 6 University of Washington, Seattle, Washington, United States; 7 Layton Alzheimer's Disease Center, Portland, Oregon, United States

## Abstract

Understanding factors that influence cognitive performance remain critical priorities, particularly among racial/ethnic groups that have higher prevalence of dementia. This study assesses race/ethnic (non-Hispanic white, non-Hispanic black, Hispanic) differences in cognitive performance in adjusted models accounting for co-existing self-reported chronic conditions (arthritis, diabetes, cancer, depressive symptoms, cardiovascular disease, hypertension, lung disease, osteoporosis, stroke), age, sex, education, and income. Data from the 2011-2017 National Health and Aging Trends Study (NHATS), a nationally-representative sample of Medicare beneficiaries (N=7,041, mean age=77.5), were used to estimate a series of cross-sectional multivariable linear regressions to evaluate race/ethnic differences in cognitive performance scores on the NHATS cognitive composite test of memory, orientation, and executive function domains (range 0-33) over seven years. In adjusted models, black participants had lower cognitive scores relative to white participants in 2011 (b=-2.25, 95% CI[-2.52, -1.98]) and by the end of the observation period in 2017 (b=-3.24, 95% CI[-3.72, -2.76]). Similarly, Hispanic participants experienced lower cognitive scores relative to white participants in 2011 (b=-2.31, 95% CI[-2.77, -1.84]) which persisted to the end of the observation window (b=-2.77, 95% CI[-3.66, -1.89]). Racial/ethnic groups had significantly lower cognitive scores relative to white Medicare beneficiaries over seven years of assessment. These analyses build toward longitudinal analyses of repeated observations of cognitive performance. Given the broad clinical and policy implications involved in caring for persons with dementia, it will be important to intervene earlier on modifiable risk factors to postpone cognitive declines among older minority ethnic adults.

